# Inhibition of tyrosine kinase receptors by SU6668 promotes abnormal stromal development at the periphery of carcinomas

**DOI:** 10.1038/sj.bjc.6605041

**Published:** 2009-04-21

**Authors:** P Farace, M Galiè, F Merigo, A Daducci, L Calderan, E Nicolato, A Degrassi, E Pesenti, A Sbarbati, P Marzola

**Affiliations:** 1 Section of Anatomy and Histology, Department of Morphological and Biomedical Sciences, University of Verona, Via Le Grazie, 8, 37134 Verona, Italy; 2 Nerviano Medical Sciences, Via Pasteur Louis, 10, 20014 Milan, Italy

**Keywords:** angiogenesis, DCE-MRI, SU6668, vascular heterogeneity, HT-29, cluster analysis

## Abstract

Dynamic contrast-enhanced (albumin-Gd-DTPA) magnetic resonance imaging, performed during 2 weeks of daily administration of an inhibitor of tyrosine kinase receptors (SU6668) in an HT-29 colon carcinoma model, revealed the onset of a hyper-enhancing rim, not observed in untreated tumours. To account for tissue heterogeneity in the quantitative analysis, we segmented tumours into three subunits automatically identified by cluster analysis of the enhancement curves using a *k*-means algorithm. Transendothelial permeability (Kps) and fractional plasma volume (fPV) were calculated in each subunit. An avascular and necrotic region, an intermediate zone and a well-vascularised periphery were reliably identified. During untreated tumour growth, the identified sub-regions did not substantially change their enhancement pattern. Treatment with SU6668 induced major changes at tumour periphery where a significant increase of Kps and fPV was observed with respect to control tumours. Histology revealed a sub-capsular layer composed of hyper-dense viable tumour cells in the periphery of untreated tumours. The rim of viable neoplastic cells was reduced in treated tumours, and replaced by loose connective tissue characterised by numerous vessels, which explains the observed hyper-enhancement. The present data show a peripheral abnormal development of cancer-associated stroma, indicative of an adaptive response to anti-angiogenic treatment.

Tumours possess an extraordinary plasticity that enables them to adapt to drastic microenvironmental changes; this leads to the undesirable development of therapy resistance, recurrence and metastatic process. In recent years, epithelial–stromal interaction in carcinomas is emerging as a crucial factor, which could be involved in their adaptive plasticity (Liotta and Kohn, 2001; Mueller and Fusenig, 2004). For example, the evolution of mammary carcinomas towards a mesenchymal phenotype in mice has been shown to be a common consequence of specific immune attack (Knutson *et al*, 2006) and drug-based treatment (Knutson *et al*, 2004). Furthermore, the spontaneous development of mesenchymal tumour after regression of the epithelial compartment has been proposed as a model of tumour recurrence (Moody *et al*, 2005).

The development of resistance to anti-angiogenic therapies is a clinically important paradigm of tumour plasticity. Clinical studies over the past two decades have demonstrated that treatment with tyrosine kinase pathway inhibitors results in a fleeting period of clinical benefit, after which the disease restart to progress. Four distinct adaptive mechanisms are currently thought to be at the basis of the transitory effects of anti-angiogenic drug (Casanovas *et al*, 2005; Bergers and Hanahan, 2008): (1) activation of alternative pro-angiogenic signaling pathways, (2) recruitment of bone-marrow-derived endothelial and blood precursors, (3) increased perycite coverage of the tumour vasculature and (4) invasion and metastasis of surrounding or distant tissues.

In the present study we provide evidence of an additional mechanism, that is the development of an aberrant vascular supporting stroma at tumour periphery, following a prolonged anti-angiogenic treatment. For this purpose, we performed a time evolution study in an experimental colon carcinoma model by dynamic contrast-enhanced magnetic resonance imaging (DCE-MRI) to assess tumour vascular responses during 2 weeks of treatment with SU6668, a small molecule inhibitor of the angiogenic receptor tyrosine kinases, VEGFR-2 (Flk-1/KDR), PDGFR-*β* and FGFR1.

## Materials and methods

The investigation complied with national legislation about the care and use of laboratory animals.

HT-29 human colon carcinoma fragments were implanted s.c. in the flanks of 12 athymic *nu/nu* mice. Treated animals (*n*=7) underwent daily administration of SU6668 (200 mg kg^−1^ per day, p.o.) for 14 days; control animals (*n*=5) received Cremophor-based vehicle. SU6668, a small molecule multi-tyrosine kinase inhibitor, is an analogue of sunitinib, developed in Sugen (La Jolla, CA, USA) and then clinically discontinued because sunitinib was preferred due to a better response.

Dynamic contrast-enhanced magnetic resonance imaging was performed at days 0, 7 and 14 on both treated and untreated animals. Gd-DTPA-albumin was used as contrast agent according to the protocol described in Marzola *et al* (2004). Three-dimensional transversal spoiled-gradient echo images were acquired with the following parameters: TR/TE=50/3.5 ms, flip angle=90°, matrix size 128 × 64 × 32, field of view 5 × 2.5 × 3 cm^3^. The acquisition time for a single scan was 104 s; a dynamic scan of 24 images was acquired with 30-s time intervals between each image (total acquisition time 53 min). The 30-s time interval allowed to avoid overheating of the gradient insert. Pre-contrast T1 values were measured using an inversion recovery snapshot Flash technique. The contrast agent was injected in bolus during the time between the first and the second scan. The plasma kinetics of contrast medium was determined *ex vivo*. From DCE-MRI data, transendothelial permeability (Kps) and fractional plasma volume (fPV) were calculated on a pixel-by-pixel basis as in Demsar *et al* (1997). To obtain mixed fPV/Kps images in red-green-blue format as in Bhujwalla *et al* (2001), fPV values were assigned to red intensities, and Kps values to green intensities.

To provide quantitative analysis taking into account of tumour heterogeneity, an automated operator-independent method, based on cluster analysis, was developed to identify sub-regions inside the tumour. A volume of interest (VOI) was manually drawn to cover the whole tumour. Each VOI was then segmented into three different compartments by applying a *k*-means cluster algorithm (implemented in Matlab; MathWorks, Natick, MA, USA) to the enhancement curves, Enh(t), defined by: 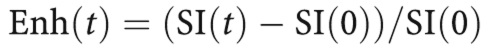 where SI(*t*) and SI(0) are the signal intensity values at time *t* and 0 respectively. The *L*_*1*_ metric (defined as *L*_*1*_(*x*, *y*)=Σ_i_ ∣*x*_i_−*y*_i_∣ for vectors *x* and *y*) was selected as the ‘distance’ function for all partitioning steps of the clustering algorithm. Although both from MRI and from histological examination two main tumour compartments were identified (an avascular core and a well-vascularised periphery), a third cluster was considered in the *k*-means clusterisation algorithm, to take into account pixels having intermediate vascularisation and/or possible partial volume effects.

For each of the three identified subunits mean Kps, fPV and volume were calculated; for the whole VOI mean and Kps and fPV were calculated. Non-parametric test (Wilcoxon) was then applied on the obtained values to statistically compare treated and untreated tumours.

At day 14 mice were killed and their tumours were excised for histological examination. Two animals belonging to the treated group were killed at day 7. After fixation in zinc fixative for 6 h, tumours were cut in half on a plane corresponding to that used for the MR images. After embedding in paraffin, 5 *μ*m thick slices were cut and stained with H&E.

## Results

### MRI analysis

**Figure 1:**
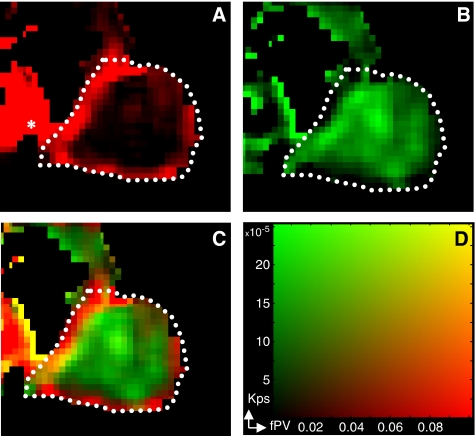
MRI mixed images. An fPV map in red (**A**) and Kps map in green (**B**) were mixed to produce the image (**C**) by the corresponding colour scale (**D**). Yellow pixels in the mixed images indicate high fPV and high Kps. In (**A**) white dotted lines represent a manually drawn region encompassing the whole tumour. The central section of the tumour is shown, and the heart is visible left of the tumour (*).

**Figure 2:**
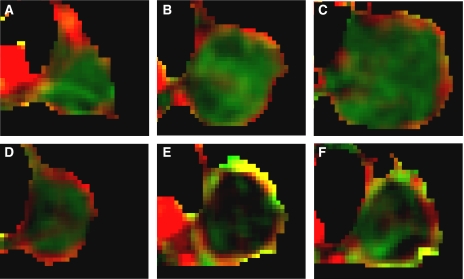
Mixed images: drug effect *vs* controls. Representative mixed images of untreated tumour growth at day 0 (**A**), day 7 (**B**) and day 14 (**C**) and of treatment progression at day 0 (**D**), day 7 (**E**) and day 14 (**F**).

The combined fPV/Kps maps clearly showed the architecture of tumour vasculature (Figures 1 and 2). Tumour periphery was characterised by high fPV (red), whereas tumour core had low fPV but detectable Kps (green). The different pattern between the periphery and the core was observed in all untreated tumours and was preserved during their growth. During SU6668 treatment, marked changes in vasculature were clearly identifiable at the tumour periphery, with the appearance of a layer of yellow pixels (high fPV and high Kps). These unexpected peripheral findings were observed in about 70% (five out of seven) of the treated tumours.

**Figure 3:**
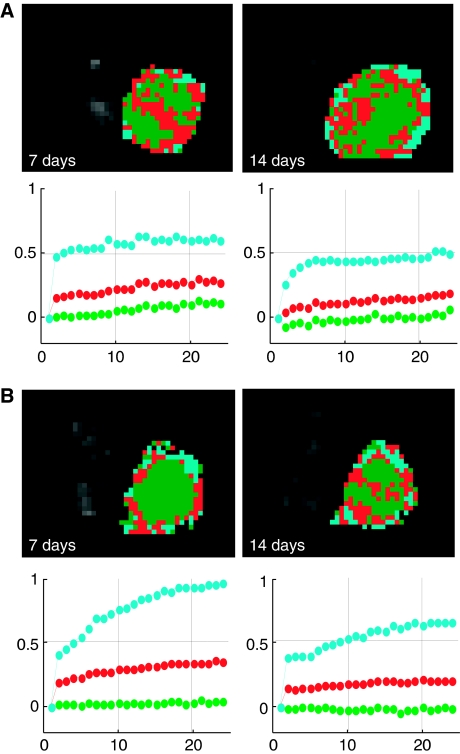
Cluster analysis automatically identifies sub-regions inside the tumour. Representative results obtained at 7 and 14 days in a control (**A**) and in a treated case (**B**) are shown. An avascular region (green), an intermediate (red) and a well-vascularised area (cyan) are identifiable. The time–course of their normalised signal enhancement is shown with the respective color identification below each picture. Number of scans is reported in abscissa. During untreated tumour growth, the identified sub-regions did not substantially change their enhancement pattern, whereas the most marked difference between untreated and treated tumours was observed in the peripheral sub-region.

**Figure 4:**
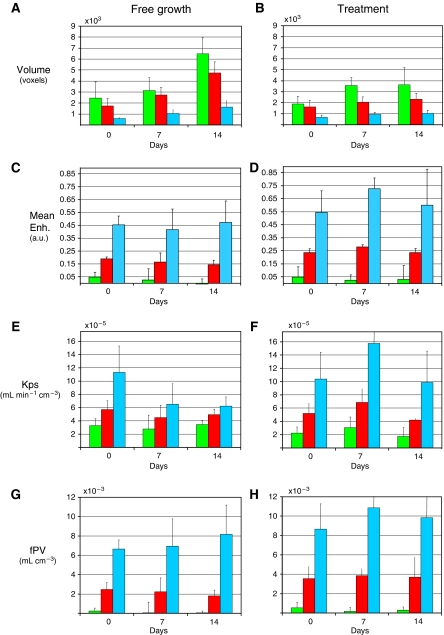
Summary of volumes and vascular parameters in the whole experiment. Volume (**A** and **B**), mean enhancement (**C** and **D**), Kps (**E** and **F**) and fPV (**G** and **H**) data on the three subunits identified by clustering algorithm, obtained by averaging data from different mice (controls, on the left; treated, on the right) at days 0, 7 and 14. Mean enhancement was taken as the mean value on the normalised enhancement curve. Error bars represent standard deviations across the group of tumours. The three identified sub-regions correspond to avascular (green), intermediate (red) and vascularised (cyan) areas.

Cluster analysis allowed identification of sub-regions inside the tumour (Figure 3), with a peripheral well-enhanced sub-region clearly identified. Full data on all three identified subunits are reported in Figure 4. The balance between the volumes of the three subunits resulted relatively stable during both untreated tumour growth (when there was an increase in total tumour volume) and anti-angiogenic administration (when there was a limited increase in total tumour volume). The most evident changes induced by SU6668 occurred in the peripheral sub-region characterised by higher vascular parameters with respect to the semi-necrotic and avascular zones. In this peripheral subunit, the progression in untreated tumour revealed a reduction in Kps; on the contrary in the treated tumours an increase was observed comparing day 7 with the pre-treatment point. In particular, Kps and fPV values were significantly higher (*P*<0.05) after 7 days of therapy than the values obtained in the corresponding control tumours (Table 1). The same table showed that mean values for the whole tumour VOI were not significantly different. At day 14 the higher Kps and fPV values in the treated tumours with respect to controls were not significant at a 0.05 level, because the MRI examination was performed only on three of the five mice that showed hyper-enhancement at day 7.

### Histological examination

**Figure 5:**
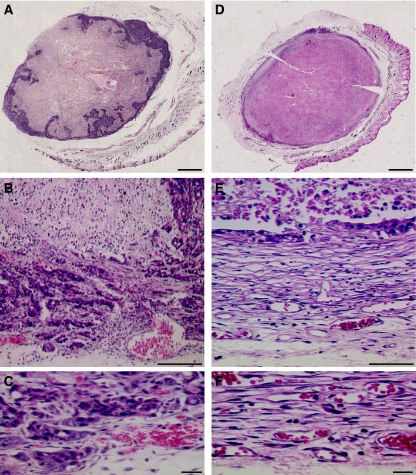
H&E histology at day 14. Histological sections from a control (**A**–**C**) and a treated (**D**–**F**) tumour. In the control case, the whole section revealed the presence of a peripheral zone with viable tumour cells showing nested intrusion into the contiguous semi-necrotic areas (**A**). Inside this matrix of viable cells, some vessels were detectable, which were particularly enlarged at the interface with tumour capsule, in a zone immediately contiguous (but external) to viable tumour tissue (**B**). At high magnification (**C**), the tumour cells appeared mixed with loose connective tissue. After 7–14 days of treatment, a marked decrease in the thickness of viable peripheral layer was clearly detectable all around the external tumour border (**D**). In the peripheral layer, the viable tumour cells appeared almost completely replaced by an increased amount of loose connective tissue (**E**), characterised by numerous vessels (**F**). Scale bar, 1.5 mm (**A** and **B**), 125 *μ*m (**C**), 60 *μ*m (**D**), 10 *μ*m (**E** and **F**).

In untreated tumours the peripheral zone, which surrounds central semi-necrotic areas, proved to be densely populated by viable neoplastic cells (Figure 5A–C). After 7–14 days of treatment (Figure 5D–F), the capsular connective tissue and the enlarged vessels, contiguous to the layer of viable tumour, were similar to those observed in control samples. However, SU6668 induced a decrease in viable tumour cells at the tumour periphery. The tumour cells were replaced by an increased amount of loose connective tissue, characterised by numerous small vessels, which presumably was responsible for the peripheral hyper-enhancement observed by DCE-MRI.

## Discussion

Dynamic contrast-enhanced magnetic resonance imaging is now being used as an *in vivo* biomarker to evaluate the efficacy of angiogenesis inhibitors and other cancer treatments (Leach *et al*, 2005; O'Connor *et al*, 2007). The potential of DCE-MRI to evaluate intra-tumoural heterogeneity and investigate its relationship with response to therapy was recently emphasised (Jackson *et al*, 2007). One approach to investigate heterogeneity is based on histogram analysis of the distribution of pharmacokinetic parameters inside the tumour, which allows to demonstrate a rim–core difference in drug effect (Checkley *et al*, 2003). To overcome the limitation of the analysis of distribution based on a single or a small number of summary parameters, statistical techniques like principal component analysis (PCA) have been proposed (O'Connor *et al*, 2005). Alternatively, clusterisation algorithms like *k*-means, closely related to PCA (Ding and He, 2004), can be used to obtain unsupervised and automatic VOI segmentation to account for tumour heterogeneity.

To develop an approach independent from any pharmacokinetics model, we have directly analysed the enhancement curves instead of using calculated Kps/fPV values. However, as the scanner gain could change between examinations, the signal intensity values were normalised to pre-contrast values. The successive evaluation of pharmacokinetic parameters on the obtained clusters, and in particular on the peripheral sub-region, proved to be more sensitive to the alteration induced by anti-angiogenic therapy than the analysis performed on the whole tumour VOI. Our results raise the hypothesis that cancer-associated stroma is involved in the ability of carcinomas to adapt to anti-angiogenic therapy. Prolonged SU6668 administration promoted abnormal development of the stromal compartment at the periphery of the treated tumours: this rim appeared significantly more perfused with respect to control tumours, consistently with the well-recognised role of stroma in tumour vasculature organisation. Our findings may suggest a different and more comprehensive mechanism of resistance to anti-angiogenic therapies, which encompasses those already described by Bergers and Hanahan (2008). In fact, an aberrant development of tumour stromal cells, including perycites, is often associated with increased release of pro-angiogenic factors, increased recruitment of bone-marrow-derived endothelial and blood precursors, and invasion and metastasis to surrounding tissues.

The observations at days 7 and 14 complement previous findings (Marzola *et al*, 2004), where the ‘standard’ effect of anti-angiogenic drugs, namely a decrease in peripheral Kps and fPV, was observed in treated tumours at early time points (24 h to 3 days of treatment). Because anti-angiogenics cannot be expected to function as effective tumour therapy on their own, regimens combining them with chemo-irradiation are essential for local tumour treatment (Timke *et al*, 2008). Anti-angiogenics induce pathophysiologic changes that can have a positive influence on tumour response to more conventional therapies (Horsman and Siemann, 2006). The appearance of an efficient vasculature and the decrease in tumour cell density, observed at the tumour periphery after prolonged SU6668 administration, could increase the sensitivity of this part of the tumour to a subsequent chemo-irradiation. On the other hand, this unexpected stromal reaction might potentially lead to an overall increase in tumour aggressiveness. Because of the termination of clinical development of SU6668 (Xiong *et al*, 2004), few data are available to verify the development of an abnormal cancer-associated stroma in human studies. However, this could be investigated with other anti-angiogenic compounds. If the described effect is confirmed in clinical research, its influence on chemo-irradiation will need to be elucidated, and should be taken into account for optimal scheduling of the combination regimen.

## Figures and Tables

**Table 1:** VOI kinetic parameters

	**Day 0**	**Day 7**	**Day 14**
*Whole tumour VOI*			
Kps 10^−5^ (mean)			
Controls	5.3±1.2	4.1±1.6	4.4±0.6
Treated	4.7±1.1	5.9±1.4	3.7±0.3
fPV 10^−3^ (mean)			
Controls	1.9±0.7	1.9±1.1	1.2±0.3
Treated	3.1±1.2	2.7±0.7	2.7±1.4
			
*Peripheral sub-region*			
Kps 10^−5^ (mean)			
Controls	11.3±3.5	6.5±2.8^*^	6.2±1.1
Treated	10.4±4.0	15.8±6.5^*^	9.9±4.7
fPV 10^−3^ (mean)			
Controls	6.7±0.8	6.9±2.6^*^	8.2±2.4
Treated	8.7±2.6	10.9±1.8^*^	9.9±5.7
% Vol. (mean)			
Controls	13.4±3.2	15.6±5.2	12.4±1.5
Treated	16.2±2.1	14.1±2.6	15.0±3.7

VOI=volume of interest; Kps=transendothelial permeability; fPV=fractional plasma volume. Mean Kps (ml min^−1^ cm^−3^) and fPV (ml cm^−3^) values obtained on the whole tumour VOI and the peripheral sub-region segmented by cluster analysis and obtained averaging on the whole tumour groups. The mean percentage volumes of the peripheral sub-region are also reported. The asterisk (^*^) indicates a significant (at a 0.05 level) difference in the comparison between treated and controls.

Data were obtained on *n*=5 control and *n*=7 treated tumours, but at day 14 only 5 of the 7 treated tumours were examined by DCE-MRI.
